# Performance Analysis of Different Backoff Algorithms for WBAN-Based Emerging Sensor Networks

**DOI:** 10.3390/s17030492

**Published:** 2017-03-02

**Authors:** Pervez Khan, Niamat Ullah, Farman Ali, Sana Ullah, Youn-Sik Hong, Ki-Young Lee, Hoon Kim

**Affiliations:** 1 Department of Electronics Engineering, Incheon National University, Incheon 406-772, Korea; pkhan@inu.ac.kr; 2 Department of Computer Science, Govt. Postgraduate Jahanzeb College, Saidu Sharif, Swat 19130, Khyber Pakhtunkhwa, Pakistan; niamatnaz@gmail.com; 3 Department of Information and Communication Engineering, Inha University, Incheon 402-751, Korea; fali@inha.edu; 4 Department of Computer and Software Technologies, University of Swat, Swat 19130, Khyber Pakhtunkhwa, Pakistan; sana@uswat.edu.pk; 5 Department of Computer Science and Engineering, Incheon National University, Incheon 406-772, Korea; yshong@inu.ac.kr; 6 Department of Information and Telecommunication Engineering, Incheon National University, Incheon 406-772, Korea; kylee@inu.ac.kr

**Keywords:** emerging sensor networks, WBAN, MAC protocols, backoff algorithm, IEEE 802.15.6, analytical model

## Abstract

The Carrier Sense Multiple Access with Collision Avoidance (CSMA/CA) procedure of IEEE 802.15.6 Medium Access Control (MAC) protocols for the Wireless Body Area Network (WBAN) use an Alternative Binary Exponential Backoff (ABEB) procedure. The backoff algorithm plays an important role to avoid collision in wireless networks. The Binary Exponential Backoff (BEB) algorithm used in different standards does not obtain the optimum performance due to enormous Contention Window (CW) gaps induced from packet collisions. Therefore, The IEEE 802.15.6 CSMA/CA has developed the ABEB procedure to avoid the large CW gaps upon each collision. However, the ABEB algorithm may lead to a high collision rate (as the CW size is incremented on every alternative collision) and poor utilization of the channel due to the gap between the subsequent CW. To minimize the gap between subsequent CW sizes, we adopted the Prioritized Fibonacci Backoff (PFB) procedure. This procedure leads to a smooth and gradual increase in the CW size, after each collision, which eventually decreases the waiting time, and the contending node can access the channel promptly with little delay; while ABEB leads to irregular and fluctuated CW values, which eventually increase collision and waiting time before a re-transmission attempt. We analytically approach this problem by employing a Markov chain to design the PFB scheme for the CSMA/CA procedure of the IEEE 80.15.6 standard. The performance of the PFB algorithm is compared against the ABEB function of WBAN CSMA/CA. The results show that the PFB procedure adopted for IEEE 802.15.6 CSMA/CA outperforms the ABEB procedure.

## 1. Introduction

Wireless Sensor Networks (WSNs) are supposed to be one of the emerging technologies of the modern era for their wide range of applications, from medical to sports, wild life and critical infrastructure monitoring, as well as military and industrial applications. In WSNs, the sensor nodes carry out distributed sensing tasks using embedded microprocessors and a tiny amount of memory [1]. The applications of WSNs are great, but typically, a WSN is meant to track and collect data through its sensor nodes. WSNs can be effectively used in healthcare monitoring systems to enhance the quality of healthcare services. For example, equipping patients with medical sensors is a fast way to monitor the patients without restricting their movements.

The rising costs of healthcare and the increase in continuous healthcare monitoring of the aging population throughout the world pose challenges for healthcare monitoring. A Wireless Body Area Network (WBAN), comprised of tiny and smart medical sensors (wearable or implanted) and a common hub, is capable of measuring, processing and forwarding important physiological data, like glucose level, heartbeats per minute, blood pressure, body temperature, oxygen level in blood and the number of breaths per minute, as well as recording, such as electrocardiograms and electromyograms. This enables health professionals to anticipate, examine and respond to critical and deleterious incidents timely [2]. The idea of a WBAN was introduced by Van Dam et al. in 2001 and since then has attracted greater research scope. The previous standards, such as IEEE 802.11, IEEE 802.11e, IEEE 802.15.4 and IEEE 802.15.3, are not appropriate for WBANs. The IEEE 802.15 Task Group 6 started to develop a communication standard in November 2007 for WBANs and was approved in February 2012, known as the IEEE 802.15.6 standard for WBANs. The standard provides short-range and low-power communication solutions.

The Medium Access Control (MAC) protocol is responsible for the access to the shared communication channel [3]. The Carrier Sense Multiple Access with Collision Avoidance (CSMA/CA) procedure of the IEEE 802.15.6 MAC protocol for WBAN uses an Alternative Binary Exponential Backoff (ABEB) procedure, and hence, the protocol varies in key features from the conventional CSMA/CA procedures adopted for IEEE 802.11, IEEE802.11e and IEEE 802.15.4. To initiate the CSMA/CA operation of the IEEE 802.15.6 MAC protocol, a Contention Window (CW) is picked as: (a) the node shall set CWto $CW_{min}$ for each newly-arrived packet; (b) an even number of failures for the same packet can only double the CW; (c) if the new CW value exceeds $CW_{max}$, the node will keep the CW at $CW_{max}$. Each idle pCCATime will lead the contending nodes to a unit lessening of their backoff counter. Moreover, any transmission on the channel during pCCATime will lead the contending nodes to lock their backoff counters until it has been idle for pSIFS. Other locking/unlocking mechanisms are beyond the scope of this study. When the backoff counter reaches zero, the contending node starts transmission [4].

IEEE 802.15.6 CSMA/CA uses the ABEB scheme to compute the backoff delay for nodes. In the ABEB algorithm, the CW for a packet retransmission is doubled only for an even number of collisions. In contention-based MAC protocols, an efficient backoff algorithm is regarded to be one of researchers’ major areas of interest. In the literature, there are many backoff algorithms, but Binary Exponential Backoff (BEB) is regarded as a standard backoff algorithm in wireless communication systems. The BEB algorithms used in different standards do not obtain the optimum performance due to enormous CW gaps induced from packet collision. Therefore, The IEEE 802.15.6 CSMA/CA has developed the ABEB procedure to avoid the large CW gaps upon each collision. However, the ABEB algorithm may lead to a high collision rate (as the CW size is incremented on every alternative collision) and poor utilization of the channel due to the unbalanced and fluctuated subsequent CW sizes. To achieve better network performance in terms of low power consumption, minimum delay and high throughput, a backoff algorithm with a gradual increase in CW is more opportune for prioritized traffic in non-saturated wireless networks. Inherently, such a backoff procedure will decrease the expected waiting time and energy consumption, and will allow the contending node to access the medium with little delay and thus, increasing the throughput performance.

In this work, we present an analytical model for the CSMA/CA mechanism of IEEE 802.15.6 employing the proposed Prioritized Fibonacci Backoff (PFB) scheme under non-saturated conditions. In our proposed model, the node state is modeled with Discrete Time Markov Chain (DTMC), and the channel state is modeled taking both homogeneous and heterogeneous networks into consideration. We concentrate on the normalized throughput, mean frame service time and energy consumption performances of the IEEE 802.15.6 CSMA/CA protocol under the PFB scheme and compare the results with the basic ABEB scheme of IEEE 802.15.6 CSMA/CA. For the homogeneous scenario, we also show that the simulation results closely follow the numerical results.

The rest of this paper is structured as follows: Section 2 presents the related studies available in the literature. Section 3 reviews an overview of the IEEE 802.15.6 standard. Section 4 describes the framework of the analytical model and performance measures. The results and discussion are conferred in Section 5, and eventually, Section 6 concludes our research findings.

## 2. Related Studies

The Binary Exponential Backoff (BEB) [5,6,7,8,9,10,11,12,13,14,15,16,17] is used widely by IEEE 802.11, WSN-based IEEE 802.15.4 and IEEE 802.15.3c MAC protocols due to its simplicity. However, some researchers in their articles have adopted the FB procedure for the CSMA/CA procedure of the IEEE 802.11 and IEEE 802.15.4 MAC protocols [18,19,20]. Even so, the conventional FB procedures cannot provide the prioritized access to the channel for a variety of user classes. To the extent of our exploration, our study is the first one that estimates the performance of IEEE 802.15.6 CSMA/CA under a different prioritized backoff algorithm. However, many researchers have investigated the ABEB procedure of the CSMA/CA protocol of the IEEE 802.15.6 standards. Performance analysis in [21] shows the effect of different access phases on the prioritized ABEB procedure of the IEEE 802.15.6 MAC protocol. In [22], numerical formulas were used to predict the theoretical performance limits of IEEE 802.15.6-based MAC protocols. They assume a collision-free network with no user priorities. In [23,24], the authors study the performance of IEEE 802.15.6 CSMA/CA, only covered by saturation conditions. The results indicate that the channel access is widely exploited by the highest User Priority (UP) nodes, while the other nodes starve. In [25], the authors present an improved adaptive MAC protocol for WBANs, where a well-defined synchronization mechanism avoids collisions. The authors in [26] propose an analytical model to evaluate the performance of a contention-based IEEE 802.15.6 CSMA/CA mechanism under saturated conditions for heterogeneous WBAN scenarios. However, in most real-world IEEE 802.15.6 networks, the saturation assumption is not likely to hold, and the traffic is mostly unsaturated. In [27], the authors examine the device lifespan performance in the contention-free period. In [28], the authors introduce a prioritized ABEB-based CSMA/CA mechanism in the Contention Access Phase (CAP) period.

We present an analytical model for the CSMA/CA mechanism of IEEE 802.15.6 employing the proposed PFB scheme under non-saturated conditions. We have taken into account the non-saturation condition where a new packet is not set up when the current packet is under service. We concentrate on the channel utilization, latency and energy consumption performances of the IEEE 802.15.6 CSMA/CA protocol under the PFB scheme and compare the results with the basic ABEB scheme of IEEE 802.15.6 CSMA/CA.

## 3. An Overview of the IEEE 802.15.6 Standard

**Figure 1:**
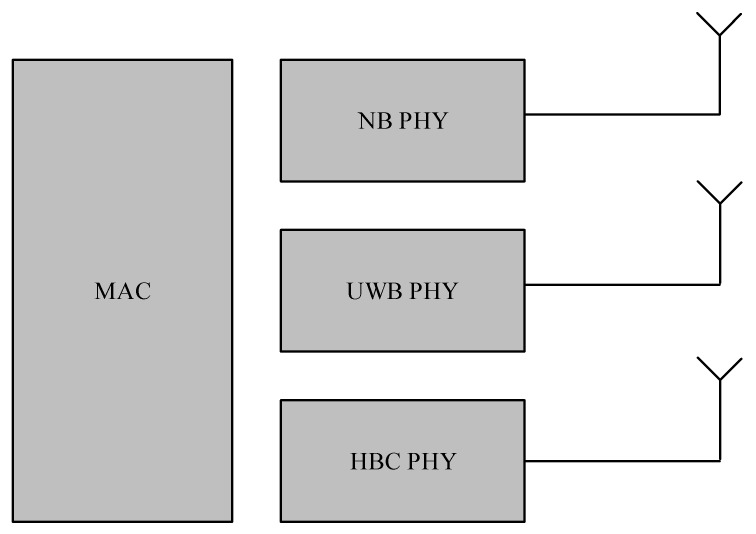
IEEE 802.15.6 PHY and MAC layers.

This section summarizes the basic attributes of the IEEE 802.15.6 Standard. We provide the insight of the IEEE 802.15.6 MAC and PHY layers. The standard defines different Physical (PHY) layers being supported by a single MAC layer, as illustrated in Figure 1. This section could be used to promptly figure out various attributes of the IEEE 802.15.6 standard and to analyze its potential for different applications of emerging body sensor networks. An absolute explanation of the PHY and MAC attributes of the IEEE 802.15.6 standard is available in [4].

### 3.1. IEEE 802.15.6 PHY Specifications

The IEEE 802.15.6 standard assists two mandatory PHYs (i.e., Human Body Communications (HBC) PHY and Ultra-Wideband (UWB) PHY) and one optional PHY (i.e., Narrowband (NB) PHY).

#### 3.1.1. NB PHY Specifications

The NB PHY is responsible for the subsequent functions [4]:

(1) Transceiver’s activation and deactivation;
(2) Clear Channel Assessment (CCA);
(3) Transmission and reception of data.

**Figure 2:**
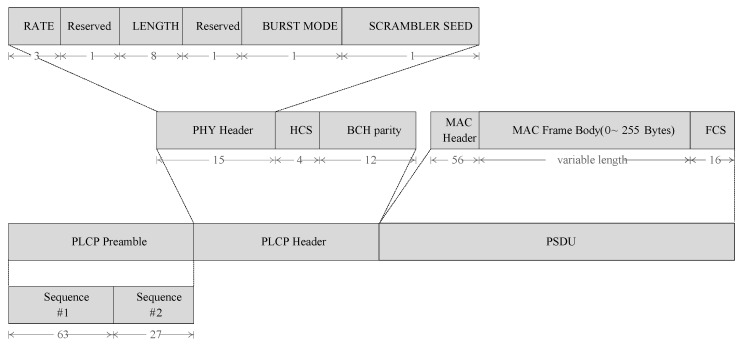
PPDU structure for NB PHY (the indicated lengths are in bits).

The NB PHY offers numerous kinds of channels (seven distinct frequency bands), modulation schemes and bit rates. The different bands are: 402–405 MHz, 420–450 MHz, 863–870 MHz, 902–928 MHz, 950–958 MHz, 2360–2400 MHz and 2400–2483.5 MHz [29]. The modulation parameters for these different bands are available in the IEEE 802.15.6 standard [4] and defined in Tables 29–35. Figure 2 shows the Physical Layer Protocol Data Unit (PPDU) structure in bits for the NB PHY.

#### 3.1.2. HBC PHY Specifications

**Figure 3:**
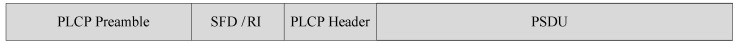
PPDU structure for HBC PHY.

The HBC PHY uses Electric Field Communication (EFC) and is operative in two bands (16 MHz and 27 MHz) to support two operation modes, i.e., high QoS mode and default mode based on the application requirements. Figure 3 shows the PPDU structure for HBC PHY.

#### 3.1.3. UWB PHY Specifications

**Figure 4:**
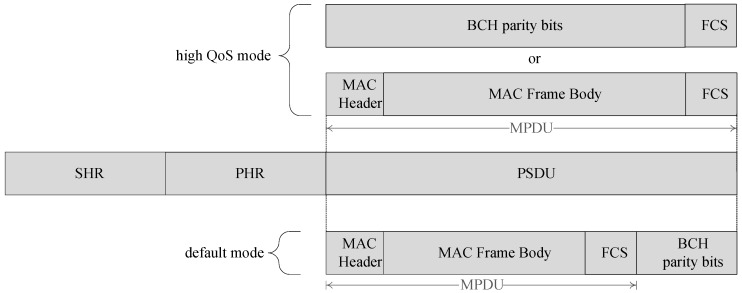
PPDU frame structure for UWB PHY.

The UWB PHY intends to actualize high performance, low complexity and power consumption, and operates in the low frequency band (Channels 0–2) and the high frequency band (Channel 3–10). As shown in Figure 4, the UWB PHY supports two operational modes, high Quality of Service (QoS) operational mode and default mode, where the first one is appointed for vital healthcare applications, and the second mode is used for medical and non-medical applications.

### 3.2. IEEE 802.15.6 MAC Specifications

In a WBAN network, the hubs and the nodes are formed into logical units, defined as Body Area Networks (BANs). A BAN may consist of only one hub and up to 64 nodes. WBAN supports one-hop and two-hop communications in a star topology. In a one-hop star topology, the exchange of frames takes place directly between the sensor nodes and the hub. In a two-hop star topology, relay-capable nodes can be used to exchange packets between the hub and the sensor nodes. A hub divides the time axis into multiple superframes. Each superframe is subdivided into allocation slots, being used for data transmission. The next sub-sections explain the communication modes, MAC format and access techniques, characterized in the IEEE 802.15.6 standard.

#### IEEE 802.15.6 Communication Modes

In IEEE 802.15.6, a hub can function in one of the subsequent three access modes;

(a) Beacon mode with beacon period superframe boundaries;
(b) Non-beacon mode with superframe;
(c) Non-beacon mode without superframe.

**Figure 5:**
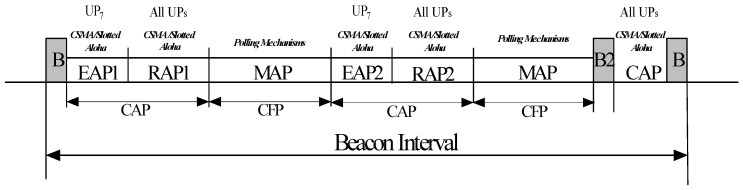
Configuration of access phases with beacon intervals.

In (a), the hub transmits a beacon on the medium at the beginning of each superframe to arrange time-referenced allocations. Figure 5 shows the superframe structure with the beacon period, which consists of various Access Phases (APs). A superframe incorporates Exclusive Access Phase 1 (EAP1) and Exclusive Access Phase 2 (EAP2) for highest priority nodes, two Random APs (RAP1 and RAP2) and a Contention Access Phase (CAP) for any kind of traffic, two Management AP (MAP) for scheduled uplink/downlink and scheduled/unscheduled bi-link allocation intervals and an optional B2 frame to announce a non-zero CAP period. Except RAP1, other APs may be assumed to be null. In (b), the hub transmits the superframe structure via Timed-Poll (T-Poll) frames and operates during MAP periods only in any superframe. In (c), the third access mode, a hub may provide Type I/II polled allocations for scheduled, unscheduled and improvised transfers. In the case of scheduled transfers, the nodes use their allocated time slots for data transmission, while in the case of unscheduled and improvised transfers, the nodes wait for a poll or post frame from the hub. Type I and Type II access phases are differentiated by the units of allocations. In Type I, the device requests allocation intervals in terms of time, while in Type II, the device requests allocation intervals in terms of the number of frames.

### 3.3. Priority Mapping

The User Priorities for accessing the medium are differentiated by eight different access categories. The type of payloads in the frame determine these prioritizing values. These traffic designations are typed as emergency or medical implant event reports, high priority medical data or network control, medical data or network control, Voice (VO), Video (VI), Excellent Effort (EE), Best Effort (BE) and Background (BK). These different data traffics are prioritized by the values of the minimum and maximum CW in the case of the CSMA/CA mechanism and Collision Probability (CP) in the slotted ALOHA case. These CW and CP bounds for CSMA/CA and slotted ALOHA, respectively, are depicted in Table 1.

### 3.4. IEEE 802.15.6 Access Mechanisms

Different access mechanisms are adopted by the IEEE 802.15.6 MAC protocol. These are divided into four categories; scheduled access, improvised and unscheduled access, random access and Medical Implant Communications Service (MICS) band access. The following sections briefly describe these access mechanisms.

#### 3.4.1. Random Access Mechanism

The IEEE 802.15.6 employs two contention-based random access mechanisms for three different PHYs. CSMA/CA with the alternative binary exponential backoff procedure can be employed for NB PHY or UWB PHY. Prioritized slotted AlOHA can be a MAC choice for UWB/HBC PHY.

##### Slotted ALOHA Protocol

**Figure 6:**
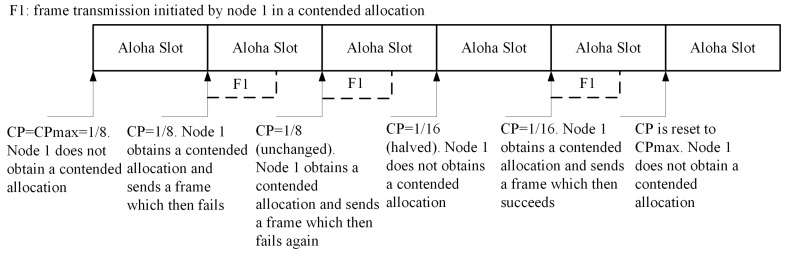
Slotted Aloha access illustration.

The IEEE 802.15.6 standard uses a particular kind of slotted Aloha as a MAC choice. In the slotted ALOHA protocol, the high and low priority traffic to access the channel are predefined by the UP values, as given in Table 1. The nodes seize contention, if z≤CP, where *z* is randomly chosen from the interval [0, 1]. To initiate the ALOHA operation of the IEEE 802.15.6 MAC protocol, a CP is picked as: (a) the node shall set CP to $CP_{max}$ for each newly-arrived packet; (b) an even number of failures for the same packet can only halve the CP; (c) if the new CP value is smaller than $CP_{min}$, the node will keep the CP at $CP_{min}$. Figure 6 illustrates an example of the slotted ALOHA operation of the IEEE 802.15.6 MAC protocol for a non-emergency node.

##### CSMA/CA Protocol

**Figure 7:**
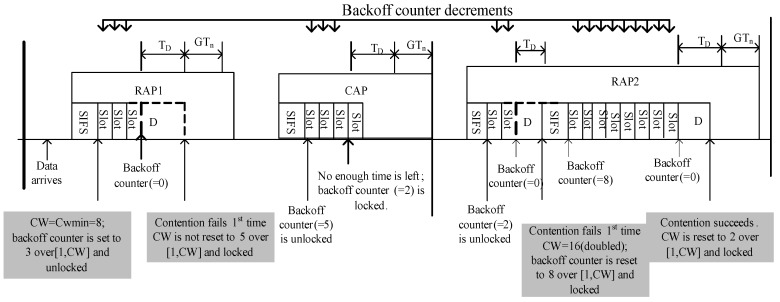
The IEEE 802.15.6 CSMA/CA mechanism.

To initiate the CSMA/CA operation of the IEEE 802.15.6 MAC protocol, a contending node having a new packet for transmission shall maintain a CW to detect a new contended allocation, where CW∈(CWmin,CWmax), and a backoff counter ∈[1,CW]. A CW is picked as: (a) the node shall set CW to $CW_{min}$ for each newly-arrived packet; (b) an even number of failures for the same packet can only double the CW; (c) if the new CW value exceeds $CW_{max}$, the node will keep the CW at $CW_{max}$. After choosing the backoff counter value between $\left[1,CW_{min}\right]$, the node starts its carrier sensing at the beginning of the next pCSMAslot to determine the current state of the channel. Each pCSMAslot has a fixed duration specified by pCSMASlotLength . The first portion of pCSMAslot corresponds to pCCATime (physical CCA) and is equal to a 63/symbol-rate in time length, while the latter portion of pCSMAslot is used by the contending node to transmit its frame to the transport medium when its backoff counter reaches zero. Each idle pCCATime will lead the contending nodes to countdown the backoff counter by one. Moreover, any transmission on the channel during pCCATime will lead the contending nodes to lock their backoff counters until they have been idle for pSIFS. Other locking/unlocking mechanisms are beyond the scope of this study. When the backoff counter reaches zero, the node then transmits. Figure 7 illustrates an example of the CSMA/CA operation of the IEEE 802.15.6 MAC protocol for a non-emergency node in the RAP period.

#### 3.4.2. Improvised and Unscheduled Access Mechanism

A hub command (poll) or management/data frame request (post) to a node can use improvised access outside/inside the scheduled, scheduled bi-link and unscheduled bi-link allocations. The unscheduled bi-link allocation adopted by the hub may exchange frames with the nodes in every superframe (one-periodic) or in every *m* superframes (m-periodic). An m-periodic bi-link allocation allows nodes to sleep between *m* superframes and thus is appropriate for low-duty cycle nodes.

#### 3.4.3. Scheduled and Scheduled-Polling Access Mechanisms

The scheduled access mechanism is used to acquire scheduled (uplink and downlink) allocations exclusively in the beacon or non-beacon mode with superframes. In addition, the scheduled polling can also adopt scheduled bi-link, polled and posted allocations, but not in non-beacon mode without superframes. All of these scheduled allocations (one-periodic or m-periodic) cannot be adopted at the same time in a single WBAN. The hub and nodes use these allocations to send/receive the management/data frames. All of the beacon periods are treated as a wakeup beacon by the nodes.

#### 3.4.4. MICS Band Communication

In the MICS band, a hub shall operate with or without superframes. The hub may choose a new channel only when required, and an implant shall communicate as a node with a hub. The hub and the node may perform unconnected mutual discovery or connected mutual discovery before their exchange of data or management type frames.

## 4. Markov Model

**Figure 8:**
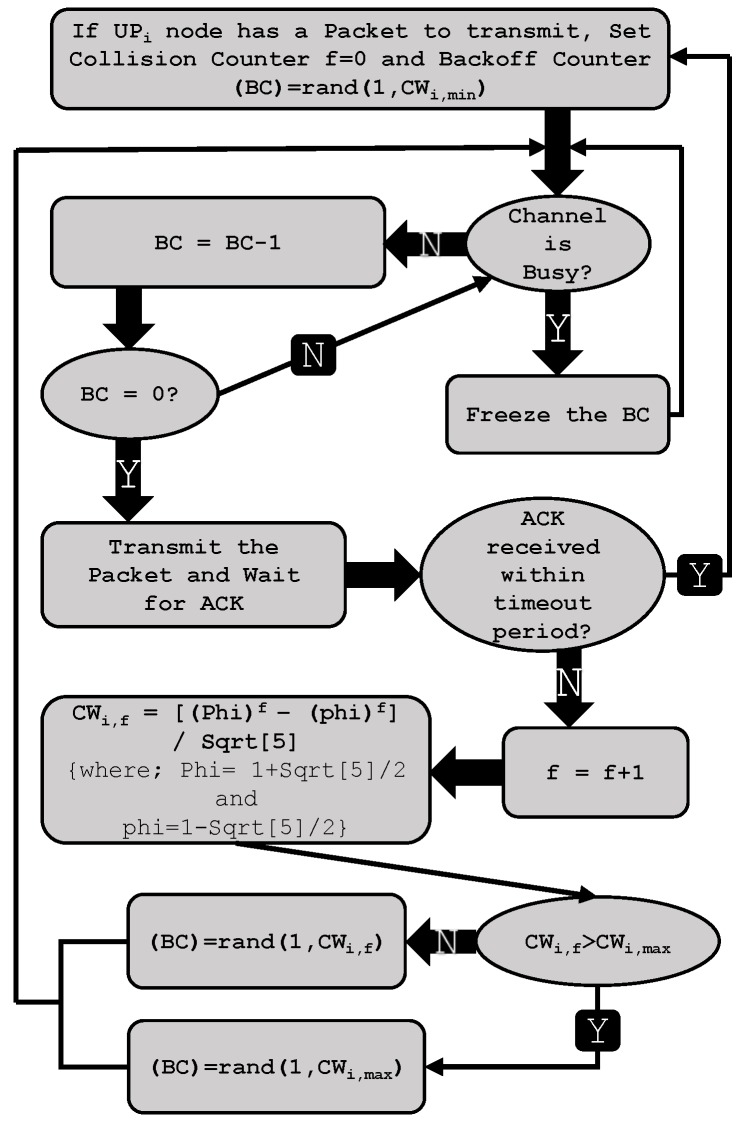
PFB-based IEEE 802.15.6 CSMA/CA procedure.

**Figure 9:**
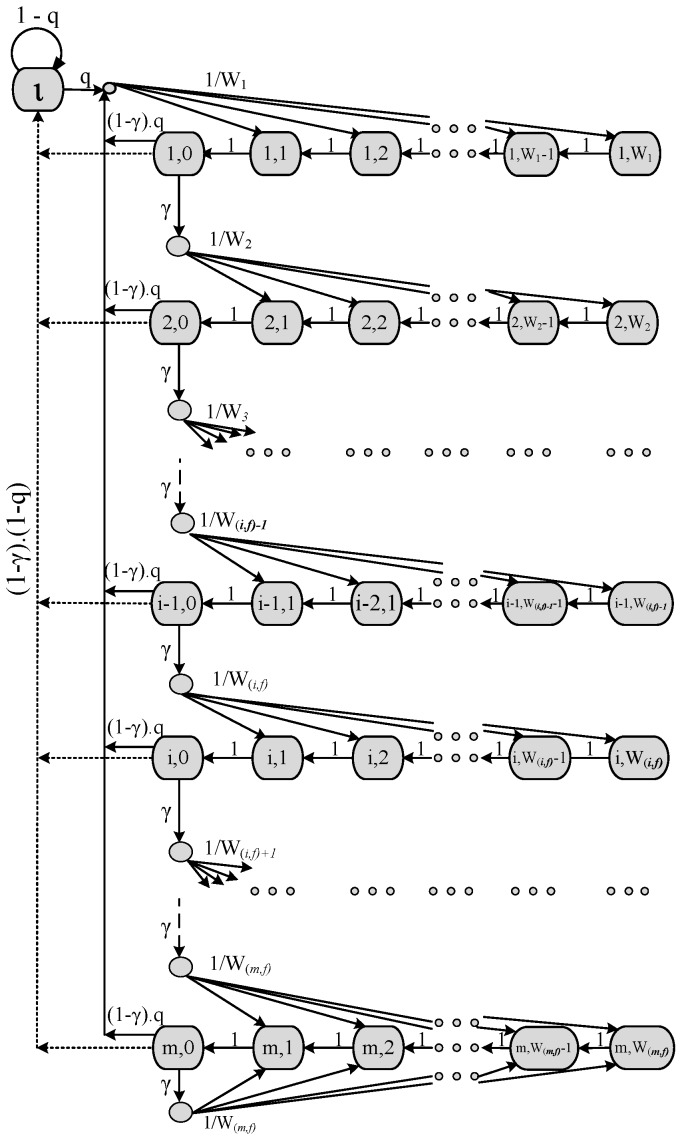
DTMC for the non-saturation behavior of the PFB-based WBAN CSMA/CA protocol.

To analyze the performance behavior of the proposed priority-based FB procedure for the IEEE 802.15.6 MAC protocol as shown in Figure 8, we develop a DTMC model as shown in Figure 9 by following Bianchi’s model [5], and compare the results with the ABEB procedure of IEEE 802.15.6 CSMA/CA under non-saturation conditions. We presume that no other packet is generated by a sensor node if it has a packet in service. The user classes, also called user priority of class *i* nodes, where i∈{0,1,2,3,4,5,6,7}, are differentiated by $CW_{min}$ and $CW_{max}$, as depicted in Table 2. The superframe structure of WBAN MAC comprises different access phases. However, we only consider activity in the RAP1 and assumed other optional APs to be null. We assume an ideal channel conditions, and a packet may not be received correctly only due to collisions. Our model considers no retry limit.

### 4.1. Solving the Discrete Time Markov Chain

The stochastic processes s(t) and b(t) depicted in Figure 9 can be modeled with a discrete time Markov chain having the following one-step transition probabilities among them:

(1) $$\left\{\begin{array}{c} Pr\left(\left(i,k-1\right)|\left(i,k\right)\right)=1,\;1\leq k\leq W_{i,f}\\ Pr\left(i+1,k\right)|\left(i,0\right)=\frac{\gamma }{W_{(i,f)+1}},\;1\leq i\leq m-1,\\ \;1\leq k\leq W_{(i,f)+1}\\ Pr\left(1,k\right)|\left(i,0\right)=q.\left(1-\gamma \right).\frac{1}{W_{1}},\;1\leq i\leq m,\\ \;1\leq k\leq W_{1}\\ Pr(l|(i,0))=(1-\gamma )(1-q),\;1\leq i\leq m\\ Pr(l|l)=1-q\\ Pr\left(\left(1,k\right)|l\right)=q.\frac{1}{W_{1}},\;1\leq k\leq W_{1}\\ Pr\left(\left(m,k\right)|\left(m,-1\right)\right)=\frac{\gamma }{W_{m,f}},\;1\leq k\leq W_{m,f} \end{array}\right.$$

The first Equation in (1) reflects the fact that, after each successful pCCAtime, the backoff counter is decremented. Upon an unsuccessful transmission, the node chooses another random backoff value uniformly distributed in the range 1,2,3,...$W_{i+1}$, and this is shown in the second transition probability of Equation (1). The third case deals with the situation that after a successful transmission, another packet is generated, and the node takes a new backoff for the new packet. The forth case models the fact that after a successful transmission, a node contains no further packet for transmission and so enters the idle state. The node remains in the idle state until a new packet arrives; the node then takes a new random backoff value in the range 1,2,3,...$W_{1}$ (the first backoff stage); these are depicted in the fifth and sixth expressions. Finally, the last case of Equation (1) views the attribute that the CW size is not increased in a subsequent packet retransmission once the $CW_{max}$ value is achieved.

With b(i,k) and b(l), we now show a closed-form solution for the Markov chain depicted in Figure 9. Let $\beta_{f}$ be the probability that a node using the PFB procedure for the IEEE 802.15.6 CSMA/CA transmits in a generic slot, regardless of the backoff stage. This probability is computed as:

(2) $$\beta_{f}=\sum_{i=1}^{m}b\left(i,0\right)$$

The stationary distributions $\sum_{k=1}^{W_{1}-1}b\left(1,k\right)+b\left(1,W_{1}\right)$ represent the topmost row of the Markov chain and are simplified as:

$$b\left(1,k\right)=\frac{W_{1}-k}{W_{1}}\times \left(1-\gamma \right)\times q\times \sum_{i=1}^{m}b\left(i,0\right)\times b\left(l\right)\times q\times \frac{W_{1}-k}{W_{1}}\times b\left(1,W_{1}\right)$$

$$b\left(1,W_{1}\right)=\frac{1}{W_{1}}\times \left(1-\gamma \right)\times q\times \sum_{i=1}^{m}b\left(i,0\right)\times b\left(l\right)\times q\times \frac{1}{W_{1}}$$

(3) $$\Rightarrow \sum_{k=1}^{W_{1}-1}b\left(1,k\right)+b\left(1,W_{1}\right)=\left(1-\gamma \right)\times \beta_{f}\times \frac{W_{1}+1}{2}$$

The stationary distributions $\sum_{k=1}^{W_{\left(m,f\right)}-1}b\left(m,k\right)+b\left(m,W_{m,f}\right)$, $\sum_{i=2}^{m-1}\sum_{k=1}^{W_{\left(i,f\right)}-1}b\left(i,k\right)$ and $\sum_{i=2}^{m-1}b\left(i,W_{i,f}\right)$ can be expressed as:

(4) $$\begin{array}{c} \sum_{k=1}^{W_{\left(m,f\right)}-1}b\left(m,k\right)+b\left(m,W_{m,f}\right)+\sum_{i=2}^{m-1}\sum_{k=1}^{W_{\left(i,f\right)}-1}b\left(i,k\right)+\sum_{i=2}^{m-1}b\left(i,W_{i,f}\right)\\ =\frac{\left(1-\gamma \right)\beta_{f}}{2}\left\{\sum_{i=1}^{m-1}\gamma^{i,f}\left(W_{i+1}+1\right)+\gamma^{m,f}\left(W_{\left(m,f\right)}+1\right)\right\} \end{array}$$

Similarly, the sum of the remaining stationary distributions of the Markov chain is given by:

(5) $$\begin{array}{c} \sum_{i=1}^{m}b\left(i,0\right)+b\left(l\right)=\beta_{f}\left\{1+\gamma +\frac{1}{q}\left(1-q\right)\left(1-\gamma \right)\right\} \end{array}$$

The stationary distribution b(l) takes into consideration the situation where the queue of the node is empty and is waiting for a packet to arrive.

To find the normalized equation,

(6) $$\sum_{i=1}^{m}\sum_{k=1}^{W_{i,f}}b\left(i,k\right)+b\left(l\right)=1$$

Let us sum the stationary distributions of (3)–(5) that give:

(7) $$\begin{array}{c} \sum_{k=1}^{W_{1}-1}b\left(1,k\right)+b\left(1,W_{1}\right)+\sum_{k=1}^{W_{\left(m,f\right)}-1}b\left(m,k\right)+b\left(m,W_{m,f}\right)+\sum_{i=2}^{m-1}\sum_{k=1}^{W_{\left(i,f\right)}-1}b\left(i,k\right)+\sum_{i=2}^{m-1}b\left(i,W_{i,f}\right)\\ +\sum_{i=1}^{m}b\left(i,0\right)+b\left(l\right)=\sum_{i=1}^{m}\sum_{k=1}^{W_{i,f}}b\left(i,k\right)+b\left(l\right)=1\;\;\;\;\;\;\;\;\;\;\;\;\;\;\;\;\;\;\;\;\;\;\;\;\;\;\;\;\;\;\;\;\;\;\;\;\;\; \end{array}$$

$$\begin{array}{c} \Rightarrow \left(1-\gamma \right)\beta_{f}.\frac{\left(W_{1}+1\right)}{2}+\frac{\left(1-\gamma \right)\beta_{f}}{2}\left\{\sum_{i=1}^{m-1}\gamma^{i,f}\left(W_{\left(i,f\right)+1}+1\right)+\gamma^{m,f}\left(W_{m,f}+1\right)\right\}\\ +\beta_{f}\left\{1+\gamma +\frac{1}{q}\left(1-q\right)\left(1-\gamma \right)\right\}=1\;\;\;\;\;\;\;\;\;\;\;\;\;\;\;\;\;\;\;\;\;\;\;\;\;\;\;\;\;\;\;\;\;\; \end{array}$$

(8) $$\Rightarrow \beta_{f}=\frac{1}{1+\gamma +\frac{1}{q}\left(1-q\right)\left(1-\gamma \right)+\left(1-\gamma \right)\sum_{i=0}^{m-1}\gamma^{i,f}\frac{W_{(i,f)+1}}{2}+\left(1-\gamma \right)\gamma^{m,f}\frac{W_{m,f}}{2}+\frac{1-\gamma^{(m,f)+1}}{2}}$$

Let $\beta_{b}$ be the normalized normalized equation for the ABEB procedure mentioned in the IEEE 802.15.6 standard, and it can be computed as:

(9) $$\Rightarrow \beta_{b}=\frac{1}{1+\gamma +\frac{1}{q}\left(1-q\right)\left(1-\gamma \right)+\left(1-\gamma \right)\sum_{i=0}^{m-1}\gamma^{i,b}\frac{W_{\left(i,b\right)}+1}{2}+\left(1-\gamma \right)\gamma^{m,b}\frac{W_{m,b}}{2}+\frac{1-\gamma^{\left(m,b\right)}+1}{2}}$$

### 4.2. Performance Metrics (Homogeneous Scenario)

We deem a single-hop star-network WBAN with *N* sensor nodes. Let (i,k) represent two random processes s(t) and b(t) for the backoff stage and backoff time counter, respectively. Under non-saturation modes, the probability of packet availability is given by $q=1-e^{-\lambda E_{state}}$, where $E_{state}$ is the expected waiting time of a $UP_{i}$ node in each state of the Markov chain and *λ* is the Poisson packet arrival rate. We compute $E_{state}$ in order to convert the states into real time.

(10) $$E_{state}=\left(1-P_{tr}\right)\times \delta +P_{tr}\times \left(1-\gamma \right)\times T_{s}+P_{tr}\times \gamma \times T_{c}$$

where *δ* represents the pCSMAslot duration, *γ* is the collision probability and $P_{tr}$ is the probability that at a minimum, one $UP_{i}$ node transmits in a given time slot and can be obtained as:

(11) $$P_{tr}=\{\begin{array}{c} 1-{\left(1-\beta_{f}\right)}^{N}\;(\mathrm{PFB}\;\mathrm{Scheme})\\ 1-{\left(1-\beta_{b}\right)}^{N}\;(\mathrm{ABEB}\;\mathrm{Scheme}) \end{array}$$

The collision probability *γ* can be expressed as follows:

(12) $$\gamma =\{\begin{array}{c} 1-{\left(1-\beta_{f}\right)}^{N-1}\;(\mathrm{PFB}\;\mathrm{Scheme})\\ 1-{\left(1-\beta_{b}\right)}^{N-1}\;(\mathrm{ABEB}\;\mathrm{Scheme}) \end{array}$$

$T_{s}$ and $T_{c}$ represent the mean time-span of a busy channel due to an acknowledged and failed transmission, respectively. $T_{s}$ and $T_{c}$ are represented as:

(13) $$\begin{array}{c} T_{s}=T_{(MAC+PHY)overhead}+T_{Payload}+T_{pSIFS}+T_{ACK}\\ T_{c}=T_{(MAC+PHY)overhead}+T_{Payload}\;\;\;\;\;\;\;\;\;\;\;\;\; \end{array}$$

Furthermore, let $P_{s}$ be the success probability of a tagged node, and it can be obtained as:

(14) $$P_{s}=\{\begin{array}{c} N\beta_{f}{\left(1-\beta_{f}\right)}^{N-1}\;(\mathrm{PFB}\;\mathrm{Scheme})\\ N\beta_{b}{\left(1-\beta_{b}\right)}^{N-1}\;(\mathrm{ABEB}\;\mathrm{Scheme}) \end{array}$$

Let Θ be the normalized network throughput, described as the fraction of time being used to transmit the actual data bits successfully. The normalized system throughput can be stated as:

(15) $$\Theta =\{\begin{array}{c} \frac{P_{s,f}\times T_{payload}}{E_{state}}\;(\mathrm{PFB}\;\mathrm{Scheme})\\ \frac{P_{s,b}\times T_{payload}}{E_{state}}\;(\mathrm{ABEB}\;\mathrm{Scheme}) \end{array}$$

$T_{payload}$ is the mean payload duration.

We are also interested in the calculation of the mean frame service time E[T], which is defined as the time span between the events that the packet reaches at the head of the queue and the time when the receiver acknowledges the packet successfully. E[T] for the PFB and ABEB procedures adopted for IEEE 802.15.6 CSMA/CA can be expressed as:

(16) $$E\left[T\right]=\{\begin{array}{c} \frac{\gamma }{1-\gamma }T_{c}+T_{s}+E_{state}\sum_{i=0}^{m-1}\gamma^{i,f}\frac{W_{f+1}}{2}+E_{state}\frac{\gamma^{m,f}W_{m,f}}{2(1-\gamma )}\;(\mathrm{PFB}\;\mathrm{Scheme})\\ \frac{\gamma }{1-\gamma }T_{c}+T_{s}+E_{state}\sum_{i=0}^{m-1}\gamma^{i,b}\frac{W_{b+1}}{2}+E_{state}\frac{\gamma^{m,b}W_{m,b}}{2(1-\gamma )}\;(\mathrm{ABEB}\;\mathrm{Scheme}) \end{array}$$

where $W_{f}$ and $W_{b}$ represent the number of backoff slots in a particular backoff stage for the Fibonacci backoff algorithm and ABEB procedure, respectively. $W_{f}$ can be computed as:

(17) $$W_{f}=\left[Phi^{f}-{\left(phi\right)}^{f}\right]/Sqrt\left[5\right]$$

where Phi=1+Sqrt[5]/2 and phi=1−Sqrt[5]/2. Whereas, for the ABEB procedure mentioned in the IEEE 802.15.6 standard, $W_{b}$ can be computed as:

(18) $$W_{b}=2^{\left\lfloor b/2\right\rfloor }CW_{min}$$

Energy is quite critical in WBANs, and therefore, we also calculate the energy consumption on a per-node per-packet basis. The expression E[T] in Equation (16) represents the time elapsed from the arrival of the packet until its successful delivery. Denoting by $P_{tx}$, $P_{rx}$, $P_{bo}$ and $P_{sleep}$ the power consumed by the transceiver of a node during transmission, reception, backoff and sleep, respectively, we derive an estimate of the energy consumption $E_{AVG}$ based on Equation (16) as follows:

(19) $$E_{AVG}=\{\begin{array}{c} \frac{1}{\lambda }\times P_{sleep}+\frac{\gamma }{1-\gamma }\times T_{c}\times P_{rx}+T_{s}\times P_{tx}+P_{bo}\times E_{state}\sum_{i=0}^{m-1}\gamma^{i,f}\frac{W_{f+1}}{2}+\\ P_{bo}\times E_{state}\frac{\gamma^{m,f}W_{\left(m,f\right)}}{2(1-\gamma )}\;(\mathrm{PFB}\;\mathrm{Scheme})\\ \frac{1}{\lambda }\times P_{sleep}+\frac{\gamma }{1-\gamma }\times T_{c}\times P_{rx}+T_{s}\times P_{tx}+P_{bo}\times E_{state}\sum_{i=0}^{m-1}\gamma^{i,b}\frac{W_{b+1}}{2}+\\ P_{bo}\times E_{state}\frac{\gamma^{m,b}W_{\left(m,b\right)}}{2(1-\gamma )}\;(\mathrm{ABEB}\;\mathrm{Scheme}) \end{array}$$

The values of $\beta_{f}$ and $\beta_{b}$ for the PFB scheme and the ABEB scheme, respectively, are derived using MATLAB to estimate the desired performance metrics.

### 4.3. Extension to Heterogeneous Scenario

Next, we will extend the performance measures of the analytical modeling for the heterogeneous scenario. The different $UP_{i}$ for WBAN, where i∈{0,1,2,3,4,5,6,7}, are differentiated by $CW_{min}$ and $CW_{max}$, as shown in Table 2. As compared to the other user classes, $UP_{7}$, being an emergency data UP class, has been given an aggressive priority. $UP_{7}$ has two types of priorities: the first is a very small contention window size, and the second is a separate access phase. As explained in Section 4, here, we do not consider the second type of priority for $UP_{7}$. The size of WBAN in terms of nodes can be computed as $N=\sum_{i=0}^{7}n_{i}$, where $n_{i}$ is the number of nodes in a $UP_{i}$. For our results, we examine three different $UP_{i}$’s with two nodes in each $UP_{i}$. We denote $\beta_{i,f}$: i∈{0,1,2,3,4,5,6,7} as the probability of transmission by a $UP_{i}$ node using the PFB procedure to model the IEEE 802.15.6 MAC protocol. This probability can be expressed as:

(20) $$\beta_{i,f}=\frac{1}{1+\gamma_{i}+\frac{1}{q}\left(1-q\right)\left(1-\gamma_{i}\right)+\left(1-\gamma_{i}\right)\sum_{j=0}^{m-1}{\gamma_{i}}^{j,f}\frac{W_{i,(j,f)}+1}{2}+\left(1-\gamma_{i}\right).\gamma_{i}^{m,f}.\frac{W_{i,(m,f)}}{2}+\frac{1-\gamma_{i}^{\left(m,f\right)}+1}{2}}$$

Let $\beta_{b}$ be the normalized equation for the ABEB procedure mentioned in the IEEE 802.15.6 standard, and it can be computed as:

(21) $$\beta_{i,b}=\frac{1}{1+\gamma_{i}+\frac{1}{q}\left(1-q\right)\left(1-\gamma_{i}\right)+\left(1-\gamma_{i}\right)\sum_{i=0}^{m-1}{\gamma_{i}}^{i,b}\frac{W_{i,(j,b)}+1}{2}+\left(1-\gamma_{i}\right).\gamma_{i}^{m,b}.\frac{W_{i,(m,b)}}{2}+\frac{1-\gamma_{i}^{\left(m,b\right)}+1}{2}}$$

where $\gamma_{i}$: i∈{0,1,2,3,4,5,6,7} is the collision probability for a class *i* node and can be obtained as:

(22) $$\gamma_{i}=\{\begin{array}{c} 1-{\left(1-\beta_{i,f}\right)}^{n_{i}-1}\prod_{j=0,j\neq i}^{7}{\left(1-\beta_{j,f}\right)}^{n_{j}}\;(\mathrm{PFB}\;\mathrm{Scheme})\\ 1-{\left(1-\beta_{i,b}\right)}^{n_{i}-1}\prod_{j=0,j\neq i}^{7}{\left(1-\beta_{j,b}\right)}^{n_{j}}\;(\mathrm{ABEB}\;\mathrm{Scheme}) \end{array}$$

The expected waiting time of a $UP_{i}$ node in each state of the Markov chain is represented by $E_{state,i}$. We compute $E_{state,i}$ in order to convert the Markov states into real time. $E_{state,i}$ can be calculated as:

(23) $$E_{state,i}=\left(1-P_{tr}\right).\delta +\sum_{i=0}^{7}P_{s,i}.T_{s}+T_{c}\left(1-\sum_{i=0}^{7}P_{s,i}\right)$$

where *δ* represents the pCSMAslot duration and $P_{tr}$ is the probability that at least one $UP_{i}$ node is transmitting in the given time slot and can be obtained as:

(24) $$P_{tr}=\{\begin{array}{c} 1-\prod_{i=0}^{7}{\left(1-\beta_{i,f}\right)}^{n_{i}}\;(\mathrm{PFB}\;\mathrm{Scheme})\\ 1-\prod_{i=0}^{7}{\left(1-\beta_{i,b}\right)}^{n_{i}}\;(\mathrm{ABEB}\;\mathrm{Scheme}) \end{array}$$

$P_{(s,f),i}$ is the success probability by a $UP_{i}$ node using the PFB scheme and can be simplified as:

(25) $$P_{(s,f),i}=n_{i}\beta_{i,f}{\left(1-\beta_{i,f}\right)}^{n_{i}-1}\prod_{j=0,j\neq i}^{7}{\left(1-\beta_{j,f}\right)}^{n_{j}}$$

Similarly, let $P_{(s,b),i}$ be the success probability of a $UP_{i}$ node using the ABEB scheme, and it can be obtained as:

(26) $$P_{(s,b),i}=n_{i}\beta_{i,b}{\left(1-\beta_{i,b}\right)}^{n_{i}-1}\prod_{j=0,j\neq i}^{7}{\left(1-\beta_{j,b}\right)}^{n_{j}}$$

The per-node throughput for a $UP_{i}$ is the fraction of time being used to transmit the actual data bits successfully. The per-class normalized throughput can be stated as:

(27) $$\Theta_{i}=\{\begin{array}{c} \frac{P_{(s,f),i}\times T_{payload}}{E_{state,i}}\;(\mathrm{PFB}\;\mathrm{Scheme})\\ \frac{P_{(s,b),i}\times T_{payload}}{E_{state,i}}\;(\mathrm{ABEB}\;\mathrm{Scheme}) \end{array}$$

$T_{payload}$ is the mean payload duration, and i=0,1,2...7.

In the case of the heterogeneous traffic scenarios, the mean frame service time of a node belonging to $UP_{i}$ can be obtained as:

(28) $$E\left[T_{i}\right]=\{\begin{array}{c} \frac{\gamma_{i}}{1-\gamma_{i}}T_{c}+T_{s}+E_{state,i}\sum_{j=0}^{m-1}\gamma_{i}^{j,f}\frac{W_{i,(j,f)+1}}{2}+E_{state,i}\frac{\gamma_{i}^{m_{i},f}W_{i,(m,f)}}{2(1-\gamma_{i})}\;(\mathrm{PFB}\;\mathrm{Scheme})\\ \frac{\gamma_{i}}{1-\gamma_{i}}T_{c}+T_{s}+E_{state,i}\sum_{j=0}^{m-1}\gamma_{i}^{j,b}\frac{W_{i,(j,b)+1}}{2}+E_{state,i}\frac{\gamma_{i}^{m_{i},b}W_{i,(m,b)}}{2(1-\gamma_{i})}\;(\mathrm{ABEB}\;\mathrm{Scheme}) \end{array}$$

where $W_{i,f}$ and $W_{i,b}$ represent the number of backoff slots in a particular backoff stage for the PFB algorithm and ABEB procedure, respectively. $W_{i,f}$ can be computed as:

(29) $$W_{i,f}=\left[{\left(Phi\right)}^{f}-{\left(phi\right)}^{f}\right]/Sqrt\left[5\right]$$

where Phi=1+Sqrt[5]/2 and phi=1−Sqrt[5]/2; whereas, for an ABEB procedure mentioned in the IEEE 802.15.6 standard, $W_{b}$ can be computed as:

(30) $$W_{i,b}=2^{\left\lfloor b/2\right\rfloor }CW_{i,min}$$

In the case of the heterogeneous traffic scenarios, the energy consumption of a node belonging to $UP_{i}$ can be obtained as:

(31) $$E_{AVG,i}=\{\begin{array}{c} \frac{1}{\lambda }\times P_{sleep}+\frac{\gamma_{i}}{1-\gamma_{i}}\times T_{c}\times P_{rx}+T_{s}\times P_{tx}+P_{bo}\times E_{state,i}\sum_{j=0}^{m-1}\gamma_{i}^{j,f}\frac{W_{i,(j,f)+1}}{2}+\\ P_{bo}\times E_{state,i}\frac{\gamma_{i}^{m_{i},f}W_{i,(m,f)}}{2(1-\gamma_{i})}\;(\mathrm{PFB}\;\mathrm{Scheme})\\ \frac{1}{\lambda }\times P_{sleep}+\frac{\gamma_{i}}{1-\gamma_{i}}\times T_{c}\times P_{rx}+T_{s}\times P_{tx}+P_{bo}\times E_{state,i}\sum_{j=0}^{m-1}\gamma_{i}^{j,b}\frac{W_{i,(j,b)+1}}{2}+\\ P_{bo}\times E_{state,i}\frac{\gamma_{i}^{m_{i},b}W_{i,(m,b)}}{2(1-\gamma_{i})}\;(\mathrm{ABEB}\;\mathrm{Scheme}) \end{array}$$

The values of $\beta_{i,f}$, $\gamma_{i,f}$ and $\beta_{i,b}$, $\gamma_{i,b}$ for the PFB scheme and ABEB scheme respectively, are derived using MATLAB to estimate the desired performance metrics.

## 5. Results and Discussion

We have compared the results of the proposed user priority-based Fibonacci backoff scheme and alternative binary exponential backoff algorithm for the IEEE 802.15.6 MAC-based CSMA/CA protocol. The PHY-dependent MAC sublayer parameters pertaining to UWB PHY are specified in the IEEE 802.15.6 standard and are used to obtain our results. For estimating energy, we used the parameters considered in [30]. These parameters are summarized in Table 3.

### 5.1. Numerical and Simulation Results for the Homogeneous Scenario

For the homogeneous scenario, we consider that all of the nodes have the same user priority in the network, i.e., all of the nodes have the same $CW_{min}$ and $CW_{max}$ values. Here, we choose three different user priority classes. The $CW_{min}$ and $CW_{max}$ values for different user priorities and UWB PHY-dependent MAC sublayer parameters are summarized in Table 2 and Table 3, respectively. We consider the $CW_{min}$ and $CW_{max}$ values of the PFB scheme in the same pattern as the ABEB procedure. The payload size is assumed to be an average size of the largest payload allowed and is equal to 1020 bits. The arrival process of packets follows a Poisson process at rates of *λ* = 0.00075 packets/μs. Moreover, to verify the proposed scheme, we have compared the numerical results with a custom-made simulation program written in the C++ programming language. We run the simulator 30 times and then take the average. For each run, the simulation time is 50 s. The simulation closely follows both backoff procedures adopted for the CSMA/CA mechanism of the IEEE 802.15.6 standard. All of the results in this section use different markers to show the simulation data, and colored lines indicate the analytical results.

**Figure 10:**
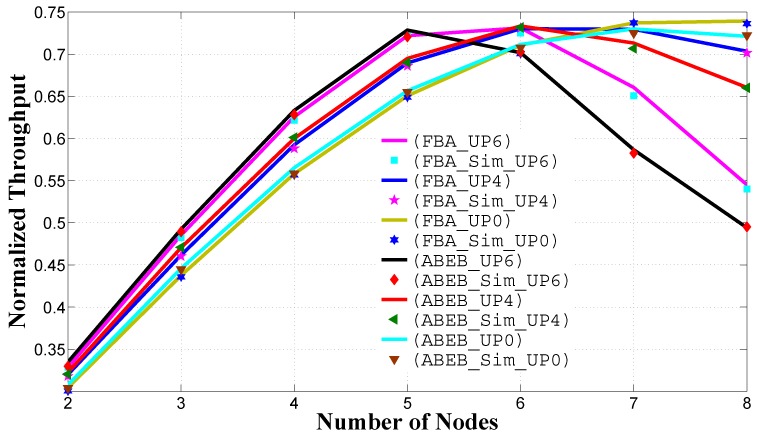
Normalized system throughput in the homogeneous case for different backoff algorithms.

**Figure 11:**
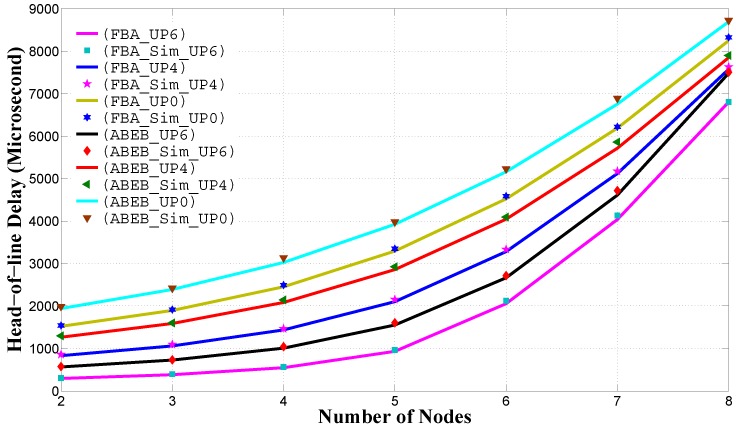
Mean frame service time in the homogeneous case for different backoff algorithms.

Figure 10 and Figure 11 show the normalized system throughput and the mean frame service time performance as a function of the number of nodes in the non-saturated homogeneous scenario, respectively. As shown in Figure 10, the PFB procedure performance metrics show an improvement over the ABEB scheme, as the network size increases. This is because we have reduced the gap between subsequent contention window sizes by adopting the PFB procedure, which subsequently reduces the waiting time, and the contending node can access the channel promptly. All of these results show that $UP_{i}$ nodes with lower $CW_{min}$ and $CW_{max}$ values can access the medium more often and, hence, can achieve a higher system throughput peak more quickly. As the number of nodes increases, we see that the throughput drastically drops for high priority nodes and slowly drops for low priority nodes, just before the saturation point. This drastic decline in the system throughput of a high priority user is due to the lower $CW_{min}$ and $CW_{max}$ values, which causes more contentions (as the number of nodes increase) and, hence, more collisions. In Figure 11, we see that the PFB procedure outperforms the ABEB procedure, as the PFB procedure leads to a smooth and gradual increase in the CW size after each collision, which eventually decreases the waiting time, and the contending node can access the channel promptly. From these results, we can optimize the $CW_{min}$ and $CW_{max}$ values to achieve better throughput with a reasonable delay.

**Figure 12:**
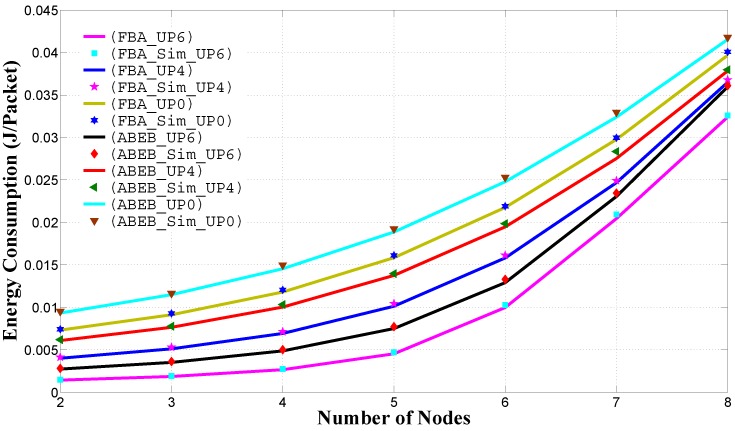
Energy consumption in the homogeneous case for different backoff algorithms.

Figure 12 shows the average energy consumption performance for the PEB and ABEB procedures, as a function of the number of nodes in the non-saturated homogeneous scenario. The average energy consumption is given by Equation (19) and calculated on a per-node per-packet basis. We see that the PFB procedure outperforms the ABEB procedure, as the PFB procedure leads to a smooth and gradual increase in the CW size after each collision, which eventually decreases the waiting time, and the contending node can access the channel promptly. Hence, the PFB decreases the expected waiting time and allows the nodes to access the medium promptly, thus improving the average energy consumption performance.

### 5.2. Numerical Results for the Heterogeneous Scenario

For the heterogeneous scenario, we consider three representative classes of users, where each class has two nodes. All of the classes have different $CW_{min}$ and $CW_{max}$. The payload size is assumed to be the average size of the largest payload allowed and is equal to 1020 bits. The arrival process of packets follows a Poisson process at a rate *λ*. The $CW_{min}$ and $CW_{max}$ values for different user priorities and UWB PHY-dependent MAC sublayer parameters are summarized in Table 2 and Table 3, respectively. We consider the $CW_{min}$ and $CW_{max}$ values of the PFB scheme in the same pattern as the ABEB procedure.

**Figure 13:**
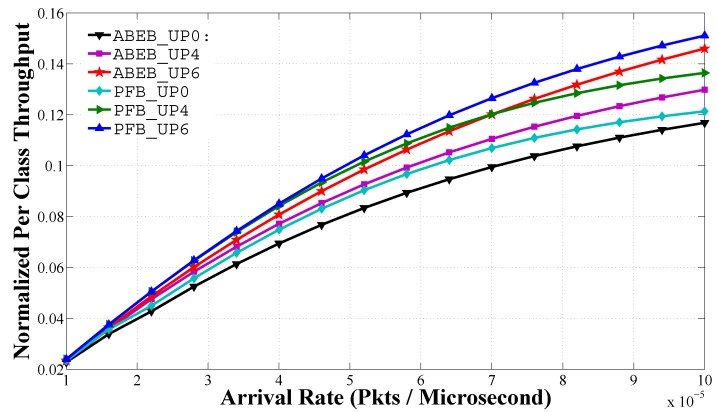
Normalized per class throughput in the heterogeneous case for different backoff algorithms.

Figure 13 shows the normalized per class throughput performance of different $UP_{i}$ nodes against the traffic load in the heterogeneous scenario. We plot the curves using different color lines and markers. All of these curves show that due to smaller values of the $CW_{min}$ and $CW_{max}$ values, high priority nodes are able to access the channel frequently. For a low arrival rate, the throughput performance gap among the user priorities is small, but as the arrival rate increases, the throughput performance gap also increases due to high traffic load. In Figure 13, the PFB procedure shows an improvement in the normalized per class throughput performance over the throughput performance of the ABEB procedure. This is due to the fact that after each collision, the PFB leads to a smooth and gradual increase in CW size, while ABEB leads to an irregular and fluctuating CW. Hence, the PFB decreases the expected waiting time and allows the $UP_{i}$ node to access the medium with little delay, thus increasing the throughput performance.

**Figure 14:**
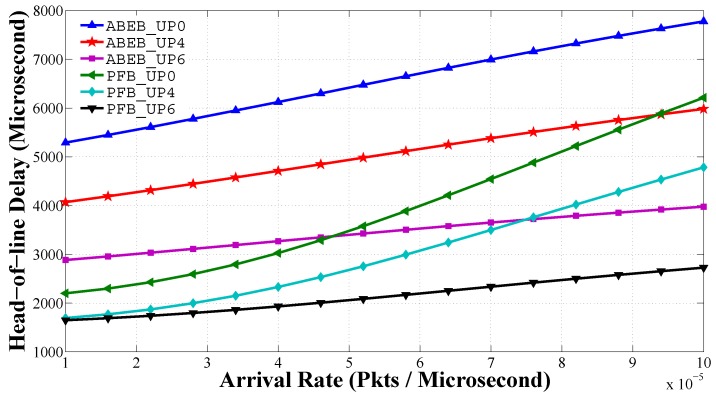
Mean frame service time of $UP_{s}$ in the heterogeneous case for different backoff algorithms.

Figure 14 shows the head of line delay performance for PEB and ABEB procedures, as a function of traffic load in non-saturated heterogeneous scenario. The head of line delay performance is given by Equation (28). For a given $UP_{i}$, we see that the mean frame service time rises with an uplift in the arrival rate. We see that $UP_{i}$ nodes with lower $CW_{min}$ and $CW_{max}$ values can access the medium more often, and hence, the delay is low. The mean frame service time increases quickly for low-priority classes than for high-priority classes as *λ* increases, because larger values of $CW_{min}$ and $CW_{max}$ introduce more average backoff time. From Figure 14, it is obvious that the proposed PFB procedure shows a reasonable difference in the delay performance over the ABEB procedure of the WBAN MAC protocol. This is due to the fact that after each collision, the PFB leads to a smooth and gradual increase in CW size, while ABEB leads to an irregular and fluctuating CW. Hence, the PFB decreases the expected waiting time and allows the $UP_{i}$ node to access the medium with little delay, thus increasing the mean frame service time performance.

**Figure 15:**
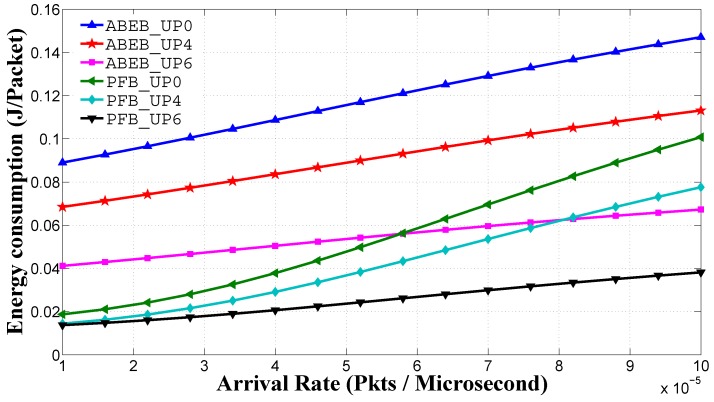
Energy consumption of $UP_{s}$ in the heterogeneous case for for different backoff algorithms.

Figure 15 shows the average energy consumption performance for the PEB and ABEB procedures, as a function of traffic load in the non-saturated heterogeneous scenario. The average energy consumption is given by Equation (31) and calculated on a per-node per-packet basis. We see that $UP_{i}$ nodes with lower $CW_{min}$ and $CW_{max}$ values can access the medium with a small backoff value, and hence, the average energy consumption is low. From Figure 15, it is obvious that the proposed PFB procedure shows a reasonable difference in the average energy consumption performance over the ABEB procedure of the WBAN MAC protocol. This is due to the fact that after each collision, the PFB leads to a smooth and gradual increase in CW size, while ABEB leads to an irregular and fluctuating CW. Hence, the PFB decreases the expected waiting time and allows the $UP_{i}$ node to access the medium promptly, thus improving the average energy consumption performance.

## 6. Conclusions

This article presents a performance comparison between the ABEB procedure of IEEE 802.15.6 CSMA/CA and the proposed PFB procedure adopted for WBAN CSMA/CA. We estimated the execution of the IEEE 802.15.6 CSMA/CA operation for both the ABEB and PFB procedures to forecast the normalized throughput, mean frame service time and energy consumption of the network by employing a Markov chain model. The numerical results show that the PFB procedure adopted for IEEE 802.15.6 CSMA/CA outperforms the ABEB procedure. This is due to the fact that after each collision, the PFB leads to a smooth and gradual increase in CW size, while ABEB leads to an irregular and fluctuating CW. Hence, the PFB procedure leads to a decrease of the waiting time, and the contending node can access the channel promptly with little delay, thus increasing the performance in terms of normalized throughput, head of line delay and energy consumption. To the extent of our exploration, our study is the first one that anticipates the implementation of IEEE 802.15.6 CSMA/CA under a different prioritized backoff algorithm. Our results also show that a smaller priority gap between the $UP_{s}$ will decrease the performance gap between the classes. We intend to extend this work to all access phases of the superframe in a noisy channel environment. We plan to fine-tune the $CW_{min}$ and $CW_{max}$ values for various priority classes, which can improve different performance metrics. A similar framework for multi-hop wireless networks can also be considered.

## Figures and Tables

**Table 1:** CW bounds for the CSMA/CA, and CP limits for slotted Aloha of WBAN.

User Priority	Traffic Designation	CSMA/CA		Slotted Aloha	
		${\mathrm{CW}}_{\min}$	${\mathrm{CW}}_{\max}$	${\mathrm{CP}}_{\max}$	${\mathrm{CP}}_{\min}$
7	Emergency or medical event report	1	4	1	$\frac{1}{4}$
6	High priority medical data or control	2	8	$\frac{1}{2}$	$\frac{3}{16}$
5	Media data of network control	4	8	$\frac{3}{8}$	$\frac{3}{16}$
4	Voice (VO)	4	16	$\frac{3}{8}$	$\frac{1}{8}$
3	Video (VI)	8	16	$\frac{1}{4}$	$\frac{1}{8}$
2	Excellent Effort (EE)	8	32	$\frac{1}{4}$	$\frac{3}{32}$
1	Best Effort (BE)	16	32	$\frac{1}{8}$	$\frac{3}{32}$
0	Background (BK)	16	64	$\frac{1}{8}$	$\frac{1}{16}$

**Table 2:** CW bounds for ABEB and PFB procedures of IEEE 802.15.6 CSMA/CA.

User Priority	ABEB		PFB	
	${\mathrm{CW}}_{\min}$	${\mathrm{CW}}_{\max}$	${\mathrm{CW}}_{\min}$	${\mathrm{CW}}_{\max}$
7	1	4	1	5
6	2	8	2	8
5	4	8	3	8
4	4	16	3	13
3	8	16	8	13
2	8	32	8	21
1	16	32	13	21
0	16	64	13	34

**Table 3:** UWB PHY-dependent MAC sublayer parameters.

Parameter	Value
Slottime	292 μs
pSIFS	75 μs
pCCA	252 μs
pCSMAMACPHYTime	40 μs
MACHeader	56 bits
MACFooter	16 bits
PHYHeader	31 bits
Payload	1020 bits
PLCPHeader(datarate)	91.9 (kb/s)
PSDU(datarate)	3159 (kb/s)
AckTime	468.4 μs
$P_{tx}$	29.9 mW
$P_{rx}$	24.5 mW
$P_{bo}$	24.5 mW
$P_{sleep}$	37 μW

## References

[1] Galzarano S., Fortino G., Liotta A. A learning-based mac for energy efficient wireless sensor networks. Proceedings of the International Conference on Internet and Distributed Computing Systems.

[2] Fortino G., Giannantonio R., Gravina R., Kuryloski P., Jafari R. (2013). Enabling effective programming and flexible management of efficient body sensor network applications. IEEE Trans. Hum.-Mach. Syst..

[3] Galzarano S., Liotta A., Fortino G. QL-MAC: A Q-learning based MAC for wireless sensor networks. Proceedings of the International Conference on Algorithms and Architectures for Parallel Processing.

[4] (2012). Part 15.6: Wireless Body Area Networks, IEEE Standard for Local and Metropolitan Area Networks.

[5] Bianchi G. (2000). Performance analysis of the IEEE 802.11 distributed coordination function. IEEE J. Sel. Areas Commun..

[6] Park C.G., Han D.H., Ahn S.J. (2006). Performance analysis of MAC layer protocols in the IEEE 802.11 wireless LAN. Telecommun. Syst..

[7] Foh C.H., Tantra J.W. (2005). Comments on IEEE 802. 11 saturation throughput analysis with freezing of backoff counters. IEEE Commun. Lett..

[8] Malone D., Duffy K., Leith D. (2007). Modeling the 802.11 distributed coordination function in nonsaturated heterogeneous conditions. IEEE/ACM Trans. Netw..

[9] Ling X., Liu K.H., Cheng Y., Shen X., Mark J.W. A novel performance model for distributed prioritized MAC protocols. Proceedings of the IEEE Global Telecommunications Conference (GLOBECOM’07).

[10] Villalón J., Cuenca P., Orozco-Barbosa L. (2007). On the capabilities of IEEE 802.11 e for multimedia communications over heterogeneous 802.11/802.11 e WLANs. Telecommun. Syst..

[11] Abu-Sharkh O.M., Tewfik A.H. (2008). Toward accurate modeling of the IEEE 802.11 e EDCA under finite load and error-prone channel. IEEE Trans. Wirel. Commun..

[12] Inan I., Keceli F., Ayanoglu E. (2009). Analysis of the 802.11 e enhanced distributed channel access function. IEEE Trans. Commun..

[13] Lauwens B., Scheers B., Van de Capelle A. (2010). Performance analysis of unslotted CSMA/CA in wireless networks. Telecommun. Syst..

[14] Pollin S., Ergen M., Ergen S., Bougard B., Der Perre L., Moerman I., Bahai A., Varaiya P., Catthoor F. (2008). Performance analysis of slotted carrier sense IEEE 802.15. 4 medium access layer. IEEE Trans. Wirel. Commun..

[15] Lee S.Y., Shin Y.S., Lee K.W., Ahn J.S. (2014). Performance analysis of Extended Non-Overlapping Binary Exponential Backoff algorithm over IEEE 802.15. 4. Telecommun. Syst..

[16] Ashrafuzzaman K., Kyung S.K. (2011). On the performance analysis of the contention access period of IEEE 802.15. 4 MAC. IEEE Commun. Lett..

[17] Pyo C.W., Harada H. (2009). Throughput analysis and improvement of hybrid multiple access in IEEE 802.15. 3c mm-wave WPAN. IEEE J. Sel. Areas Commun..

[18] Shurman M.M., Al-Mistarihi M.F., Alomari Z.A. MAC layer back-off algorithm for ad hoc networks. Proceedings of the 36th International Convention on Information & Communication Technology Electronics & Microelectronics (MIPRO).

[19] Yassein M.B., Al Oqaily O., Min G., Mardini W., Khamayseh Y., Manaseer S.S. Enhanced Fibonacci Backoff Algorithm for Mobile Ad-Hoc Network. Proceedings of the IEEE 10th International Conference on Computer and Information Technology (CIT).

[20] Tashtoush Y., Darwish O., Hayajneh M. (2014). Fibonacci sequence based multipath load balancing approach for mobile ad hoc networks. Ad Hoc Netw..

[21] Khan P., Ullah N., Alam M.N., Kwak K.S. (2015). Performance analysis of WBAN MAC protocol under different access periods. Int. J. Distrib. Sens. Netw..

[22] Ullah S., Kwak K.S. Throughput and delay limits of IEEE 802.15. 6. Proceedings of the IEEE Wireless Communications and Networking Conference (WCNC).

[23] Rashwand S., Misic J. Performance evaluation of IEEE 802.15. 6 under non-saturation condition. Proceedings of the IEEE Global Telecommunications Conference (GLOBECOM).

[24] Rashwand S., Mišić J., Khazaei H. (2011). IEEE 802.15. 6 under saturation: Some problems to be expected. J. Commun. Netw..

[25] Ahmad A., Javaid N., Khan Z.A., Imran M., Alnuem M. iA-MAC: Improved adaptive medium access control protocol for wireless body area networks. Proceedings of the 14th International Symposium on Communications and Information Technologies (ISCIT).

[26] Rashwand S., MišIć J. (2012). Effects of access phases lengths on performance of IEEE 802.15. 6 CSMA/CA. Comput. Netw..

[27] Tachtatzis C., Di Franco F., Tracey D.C., Timmons N.F., Morrison J. An energy analysis of IEEE 802.15. 6 scheduled access modes. Proceedings of the 2010 IEEE Globecom Workshops.

[28] Ullah S., Imran M., Alnuem M. (2014). A hybrid and secure priority-guaranteed MAC protocol for wireless body area network. Int. J. Distrib. Sens. Netw..

[29] Chávez-Santiago R., Sayrafian-Pour K., Khaleghi A., Takizawa K., Wang J., Balasingham I., Li H.B. (2013). Propagation models for IEEE 802.15. 6 standardization of implant communication in body area networks. IEEE Commun. Mag..

[30] Ullah N., Khan P., Kwak K.S. (2011). A Very Low Power MAC (VLPM) protocol for wireless body area networks. Sensors.

